# Head and Neck Cancer (HNC) Prehabilitation: Advantages and Limitations

**DOI:** 10.3390/jcm13206176

**Published:** 2024-10-17

**Authors:** Sara Demurtas, Hellas Cena, Marco Benazzo, Paola Gabanelli, Simone Porcelli, Lorenzo Preda, Chandra Bortolotto, Giulia Bertino, Simone Mauramati, Maria Vittoria Veneroni, Ester Orlandi, Anna Maria Camarda, Nagaia Madini, Chiara Annamaria Raso, Laura Deborah Locati

**Affiliations:** 1 Internal Medicine and Therapeutics Department, University of Pavia, 27100 Pavia, Italy; chiaraannamari.raso01@universitadipavia.it (C.A.R.);; 2 Unit of Medical Oncology, Maugeri Clinical Research Institutes IRCCS, 27100 Pavia, Italy; 3 Laboratory of Dietetics and Clinical Nutrition, Department of Public Health, Experimental and Forensic Medicine, University of Pavia, 27100 Pavia, Italy; hellas.cena@unipv.it (H.C.); nagaia.madini@unipv.it (N.M.); 4 Unit of Clinical Nutrition, Maugeri Clinical Research Institutes IRCCS, 27100 Pavia, Italy; 5 Department of Otorhinolaryngology, University of Pavia, 27100 Pavia, Italy; marco.benazzo@unipv.it; 6 Department of Otolaryngology-Head and Neck Surgery, IRCCS Policlinico San Matteo Foundation, 27100 Pavia, Italy; g.bertino@smatteo.pv.it (G.B.); s.mauramati@smatteo.pv.it (S.M.); mv.veneroni@smatteo.pv.it (M.V.V.); 7 Unit of Psychology, Maugeri Clinical Research Institutes IRCCS, 27100 Pavia, Italy; paola.gabanelli@icsmaugeri.it; 8 Department of Molecular Medicine, University of Pavia, 27100 Pavia, Italy; simone.porcelli@unipv.it; 9 San Matteo Clinic IRCCS Foundation, 27100 Pavia, Italy; 10 Diagnostic Imaging and Radiotherapy Unit, Department of Clinical, Surgical, Diagnostic, and Pediatric Sciences, University of Pavia, 27100 Pavia, Italy; lorenzo.preda@unipv.it (L.P.); chandra.bortolotto@unipv.it (C.B.); 11 Radiology Institute, Fondazione IRCCS Policlinico San Matteo, 27100 Pavia, Italy; 12 Department of Clinical, Surgical, Diagnostic, and Pediatric Sciences, University of Pavia, 27100 Pavia, Italy; ester.orlandi@cnao.it; 13 Radiation Oncology Unit, CNAO National Center for Oncological Hadrontherapy, 27100 Pavia, Italy; annamaria.camarda@cnao.it

**Keywords:** head and neck cancer, prehabilitation, physical exercise, nutritional intervention, psychological support

## Abstract

Cancer prehabilitation is the process between the time of cancer diagnosis and the beginning of the active acute treatment; prehabilitation consists of various need-based interventions, e.g., physical activity, a nutritional program, and psychological support. It can be delivered as unimodal or multimodal interventions. Physical activity, including resistant exercise and aerobic activities, has to be tailored according to the patient’s characteristics; nutritional support is aimed at preventing malnutrition and sarcopenia; while psychological intervention intercepts the patient’s distress and supports specific intervention to address it. In addition, multimodal prehabilitation could have a potential impact on the immune system, globally reducing the inflammatory processes and, as a consequence, influencing cancer progression. However, many challenges are still to be addressed, foremost among them the feasibility of prehabilitation programs, the lack of adequate facilities for these programs’ implementation, and the fact that not all prehabilitation interventions are reimbursed by the national health system.

## 1. Introduction

### Definition of Prehabilitation in Cancer Patient Trajectory

Cancer prehabilitation is defined as the process on the continuum of care which occurs between the time of cancer diagnosis and the beginning of the active acute treatment [1]. Prehabilitation aims at strengthening physical, emotional, and cognitive health before undergoing a cancer treatment, having as its main objectives the prevention and reduction of treatment-related morbidities and mortality [1]. In addition, prehabilitation seems to be effective in ameliorating the adverse events of cancer therapies, even when performed during active treatment and, therefore, it could be applied to different phases of a cancer patient’s journey [2]. Prehabilitation may include unimodal and multimodal interventions. In the first case, it focuses on a single intervention, mainly physical exercise; on the contrary, multimodal prehabilitation consists of various need-based interventions, three major topics of which are physical activity, a nutritional program, and psychological support. However, no consensus has been reached about the scope and the correct interventions of prehabilitation. Indeed, it may have different objectives according to the type of oncological treatment. For example, for those patients who are candidates for surgical interventions, outcomes may include the reduction of perioperative and postoperative morbidities, such as the incidence of wound infections and venous thromboembolism, the reduction of hospital length of stay, and hospital readmissions [3]. When a patient is a candidate for chemo- and radiotherapy, possible outcomes could be the improvement of the patient’s adherence to the scheduled therapy, reduction of treatment-related adverse events, and amelioration of their health-related quality of life (HRQoL) [4]. Functional capacity is the most common outcome in prehabilitation programs: it corresponds to the physical and mental capability to perform necessary or desirable tasks and activities of daily living; this allows the individual to face stressful events, such as cancer diagnosis and its treatment [5].

Head and neck (HNC) are rare cancers, accounting worldwide for 890,000 new cases in 2017, with 52,000 cases attributable to HPV infection [6]. Indeed, HPV-unrelated and HPV-related tumors are characterized by different risk factors, biological profile, and clinical outcome, with similar morbidity and side effects burden, derived from the complex therapeutic approaches [7]. Evidences of prehabilitation in HNC patients are limited at the moment to surgical settings. Our purpose is to perform a narrative review on prehabilitation in HNC, to explore the potential benefits, and to investigate how multimodal programs of prehabilitation may impact on patients’ outcomes. Finally, we will discuss the barriers limiting the development of prehabilitation in clinical practice.

## 2. Methods

Trials and articles were collected by comprehensive PubMed/MEDLINE database searching, which was concluded on 27 April 2024. Research keywords and phrases included those related to the head and neck anatomical region (e.g., oral cavity, oropharynx, hypopharynx, larynx, head and neck), multimodal prehabilitation (e.g., prehabilitation, physical activity, physical exercise, nutritional intervention, nutritional counseling, psychological support), type of treatment (e.g., surgery, radiotherapy, radiation therapy, chemoradiotherapy, chemoradiation) and cancer (e.g., tumor, cancer); these were combined through the “AND” operator. Randomized control trials and systematic reviews were included; feasibility studies, literature reviews, and abstract-only text were excluded.

## 3. Relevant Sections

### 3.1. Evidence of Prehabilitation in Head and Neck Cancer

#### 3.1.1. Surgery

The surgical management of HNC presents a multifaceted challenge, often involving surgical interventions that can be physically and emotionally demanding for patients. Among the challenges faced, there are weight loss and dysphagia, which not only affect quality of life (QoL) but also impact on surgical outcomes. Recognizing these challenges, prehabilitation has emerged as a potential strategy to optimize patient health before surgery, with the goal of improving postoperative recovery and outcomes.

In a recent comprehensive meta-analysis conducted by Seth et al. [8] and including 31 articles, prehabilitation significantly decreased mortality rate, serious complication rate, dysphagia rate, and length of hospital stay (LOS). In detail, physical exercise combined with nutritional support resulted in a significantly lower rate of complications (RR 0.66; 0.49, 0.88, *p* < 0.005); moreover, nutritional support and psychoeducation resulted in a significant reduction in mortality risk by 38%. This suggests that pre-surgical interventions hold promise in fortifying patients’ physiological resilience, thereby mitigating the likelihood of adverse events both during and following surgical procedures.

In addition, a correlation between psychoeducation interventions and the reduction in LOS following surgery was reported [9], also suggesting a short-term benefit. The potential mechanism lies in psychoeducation, fostering patient adherence to treatment regimens, and cultivating a positive mindset post-treatment.

Most of the previous research projects about exercise prehabilitation were aimed at preventing or reducing dysphagia or swallowing dysfunction [10,11]. Indeed, the surgical disruption of structures involved in the swallowing reflex as well as radiotherapy (RT) alone or combined with chemoradiation (CRT) can cause dysphagia, leading to a reduction in appetite and weight loss [12]. However, investigations revealed divergent effects of distinct prehabilitation modalities on dysphagia management [8,10]. While exercise-based interventions (especially swallowing exercises according to the University of Texas [13]) demonstrated notable improvements in dysphagia outcomes [8,14], nutrition-based approaches did not yield significant benefits in this regard. This fact highlights the importance of tailored interventions targeting the specific mechanisms underlying dysphagia, with exercise playing a crucial role in neuromuscular rehabilitation [10].

However, despite the persistence of dysphagia-related challenges, early nutritional interventions were effective in mitigating weight loss, underscoring the importance of proactive nutritional support in the preoperative period [8,15,16]. It is widely acknowledged in the literature that nutritional supplements containing essential amino acids such as arginine and glutamine, which are not endogenously synthesized by the body, play a vital role in boosting the immune system and facilitating tissue repair, particularly in post-traumatic conditions [16]. However, it is crucial to acknowledge the multifactorial nature of weight loss in HNC, including the mechanical dysphagia secondary to the primary tumor site and the side effects (e.g., stomatitis) related to treatment. This aspect highlights the need for comprehensive and multidisciplinary approaches to address this issue effectively.

#### 3.1.2. Chemotherapy and Radiotherapy

There are several treatment options for locally advanced HNC, including surgery combined with adjuvant RT plus or minus chemotherapy (CT) or CRT, depending on the cancer stage, site, and pathological risk factors [17]. Intensity-modulated radiotherapy (IMRT) signified a major improvement over conventional 2D and 3D RT in the last 20 years. IMRT allows the delivery of a higher dose to specified target volumes, while reducing the dose to adjacent organs at risk (OARs). The definitions of dose-volume response and radiation-dose constraints for salivary glands and constrictor muscles have been well established. In clinical practice, this has had a direct impact on the acute and late toxicity burden, especially on those toxicities such as xerostomia and dysphagia that seriously affect the patient’s quality of life [18]. In addition, trends favoring IMRT over the oldest RT techniques for overall survival have been reported, without significant impact on outcome [19]. CRT is associated with a significant incidence of grade 3 acute (83%) and late (35%) treatment-related morbidity [20]. Adverse events such as fatigue, dysphagia, and weight loss could seriously affect patients’ QoL and performance status, and result in a reduced adherence to CRT, frequent treatment interruptions, CRT dose reduction and/or definitive treatment withdrawn, which globally means a worsening of survival outcomes [21,22,23,24]. Frequently, HNC patients are heavy smokers and alcohol consumers with poor oral hygiene. The prevention of oral and dental health complications is mandatory to avoid any treatment interruption and late toxicities, such as osteoradionecrosis of the jaw. An orthopantomography evaluation, or a dental CT scan in the most complex cases, with a dentist visit, are suggested before starting CRT. This would prepare the oral cavity for CRT or for surgical intervention. Dental restoration is recommended with accurate teeth cleaning to reduce the oral bacterial load. Xerostomia may contribute to teeth decay as well. This is a late side effect, related to the irradiation of salivary glands. Severe xerostomia observed with 2D RT has been overcome with the use of more tailored RT techniques, such as 3D and IMRT. Despite different attempts during the years, the radioprotective agents investigated (e.g., pilocarpine) [25] failed to demonstrate a significant benefit, with the exception of amifostine, which intravenous administration and its toxic side effects such as hypotension have severely limited its further application in clinic [26]. Multimodal prehabilitation interventions might be useful in this perspective, acting to prevent or reduce these morbidities.

Evidence supporting prehabilitation programs in the CRT setting are limited. In fact, randomized control trials are few, with heterogenous patient populations and interventions that do not always rely on multimodal prehabilitation strategies.

Most of the trials are focused on improving swallowing function and, consequently, dysphagia or optimizing nutritional status, even if the results are contradictory. Targeted prophylactic swallowing exercises (the Effortful Swallow [27], two tongue base retraction exercises [28,29], the Super Supraglottic Swallow technique [30], and the Mendelssohn maneuver [31]) during CRT seem to improve the swallowing function at 3 and 6 months from CRT, while no benefit was observed at the end of CRT, nor 9 and 12 months after, maybe due to a lack of statistical power [32]. In addition, swallowing exercises were associated with less gastrostomy tube dependence at the end of CRT and after 3 months [33]. At the opposite, some trials did not demonstrate any impact on dysphagia outcomes. Forty-four HNC patients who were candidates for curative RT were randomized to perform swallowing exercises at home or to the standard of care; adherence to these exercises was not good, diminishing with time after RT, and dropouts were frequent due especially to fatigue, equally divided between the two arms [34]. A more recent study conducted on 240 HNC patients eligible for curative RT has shown the significant effect of swallowing exercises and physiotherapy-led progressive resistance training (PRT) on long-term mouth opening, QoL, depression, and anxiety compared to usual care; however, the trial has not reached its primary endpoint of improving swallowing safety [35]. One additional study in 66 patients with HNC treated with RT with or without CT evaluated the effect of TheraBite^®^ (a portable device utilizing repetitive passive motion and stretching of jaw musculature) on preventing trismus but did not show significant differences between intervention and the control group [36].

Some studies demonstrated that combined physical activity (moderate-intensity aerobic, resistance, and flexibility exercises) during CRT is feasible and that this is associated with an attenuated worsening or an improvement in physical fitness (e.g., body composition and muscle strength) and HRQoL endpoints measured with the European Organization for Research and Treatment of Cancer Quality of Life Core Questionnaire [37] (EORTC QLQ-C30) and the head and neck module [38] (EORTC-QLQ-H&N35) [39,40]. A larger randomized trial conducted on 148 patients who were all candidates for radical CRT for HNC confirmed that an exercise program of aerobic and active resistance exercises significantly improves functional capacity, QoL, and prevention of worsening of fatigue [41].

Other pilot randomized control trials have investigated the effects of combining physical, nutritional, and lifestyle interventions, oral nutritional supplements (ONS), and nutritional screening and support in patients undergoing CRT [42,43]. They have confirmed that prehabilitation strategies are feasible and have shown that they could mitigate the loss of muscle mass, but do not significantly influence the body composition, while having a positive effect on some fitness (e.g., total grip strength, sit-to-stand and sit-and-reach scores), QoL, and nutrition status outcomes. Some studies have investigated the impact of nutritional interventions alone. One trial has demonstrated that providing early nutritional counseling to patients who are candidates for CRT, irrespective of nutritional status, is essential for improving treatment adherence and survival outcome [15]. Another study has shown that the use of ONS in addition to nutritional counseling is associated with improvement in weight maintenance, protein–calorie intake, and global QoL; furthermore, adding ONS seemed to reduce CT and/or RT dose reduction or complete suspension [44]. However, an intensive nutritional care program, consisting of individualized nutritional counseling, did not improve QoL scores, weight maintenance, or energy and protein intakes in another randomized trial [45].

The trials mentioned above are listed in Table 1; for each of them, the type of intervention, the study population, the primary and secondary objectives, and the results have been described.

Nevertheless, trial populations (e.g., patients who were candidates for exclusive RT, or CRT or RT with or without CT), interventions (e.g., different types of physical exercises, physical exercises alone or in combination with nutritional intervention) and efficacy outcomes (e.g., outcomes related to swallowing function, physical fitness, and HRQoL) were really heterogenous within and between studies. More evidence is needed to correctly define the implementation of multimodal prehabilitation programs in HNC patients’ journies. In addition, no randomized clinical trials evaluating the role of prehabilitation in recurrent and/or metastatic HNC patients who were candidates for palliative systemic treatment have been conducted to date.

### 3.2. The Potential Contribution of Multimodal Prehabilitation in HNC

**Figure 1:**
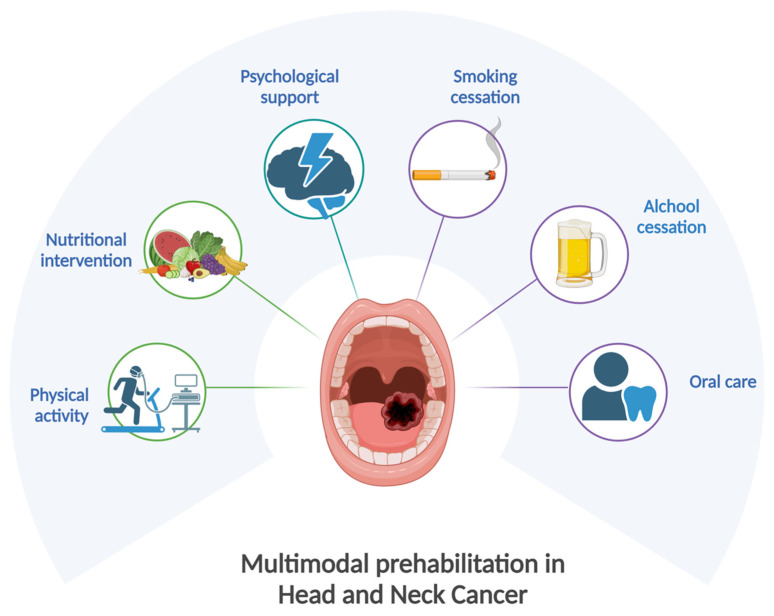
Multimodal prehabilitation in HNC patients. Created with BioRender.com.

In daily clinical practice, HNC patients are already submitted to a sort of prehabilitation (Figure 1). Indeed, they are prepared by the multidisciplinary team for the treatment, either surgery or CRT, with behavioral interventions for smoking and alcohol cessation, odontostomatology examination, and nutritional screening. These interventions are generally provided in order to reduce treatment-related side effects and comorbidities, improving the patient’s outcome. However, procedures are not standardized, and they would need to be systematized. The timely initiation of therapy is crucial for optimal clinical outcomes. However, in everyday clinical practice, the interval between diagnosis and the start of cancer treatment already involves a series of essential procedures, including multidisciplinary team evaluation, radiological assessment, central venous access placement, and dental and nutritional assessment as discussed above. All these procedures require a certain amount of time, which can be appropriately used to implement a prehabilitation program without delaying the initiation of definitive cancer treatment. Patients’ selection is crucial, and those with advanced tumors requiring a rapid initiation of the treatments are not the best candidates for prehabilitation.

#### 3.2.1. Physical Activity

There is still little known about the effects of exercise prehabilitation in HNC patients and, to the best of our knowledge, there is only one trial on exercise prehabilitation for people diagnosed with HNC undergoing surgery [48]. In the absence of scientific evidence, general guidelines can be suggested for designing an effective exercise prehabilitation intervention. Physical activity interventions should include resistance exercise to gain muscle strength, reduce fatigue, and limit the loss of muscle mass. Additionally, HNC patients should be invited to regularly practice aerobic exercise such as cycling and walking to preserve cardio-respiratory fitness, mental health, immune function, and overall functional capacity. Given the lack of evidence about the most beneficial approach, we cannot provide specific recommendations in terms of exercise types and methods. However, exercise programs should be prescribed by a skilled physician, for example a sports medicine doctor, according to individual needs and, when possible, should include a combination between supervised exercise sessions and home-based activities, self-managed or exploiting the use of telemedicine facilities monitoring. A battery of functional tests or laboratory evaluations such as neuromuscular function and cardiopulmonary exercise tests should be provided, as an essential step of the comprehensive care plan, to collect baseline data and optimize outcomes. The time required to generate health benefits and the question of how to quantify these benefits are also important determinants that should be considered, even in relation to the relatively short time frame available between the diagnosis and the start of standard therapeutic procedures. Finally, prehabilitation exercise should also aim at educating patients about the importance of being more physically active during the hospitalization and recovery because it has been demonstrated that physical exercise enhances patients’ ability to cope with the challenges of cancer treatment. Indeed, ASCO guidelines recommended aerobic and resistance exercise not only before starting therapies but even during active treatment with curative intent to mitigate the side effects associated with therapy [2]. This recommendation provides a new point of view in respect to physical activity exercise during oncological treatments.

#### 3.2.2. The Importance of Nutritional Status and Nutritional Support

In the realm of HNC prehabilitation, the role of nutritional status and support emerges as a pivotal factor in enhancing patient outcomes. The prevalence of malnutrition among HNC patients is alarmingly high, primarily due to challenges such as dysphagia, altered taste, and reduced appetite, which are inherent to the disease [49], considering the involvement of the upper part of the aerodigestive tract in most cases. This malnutrition significantly impairs treatment efficacy, and there is a direct correlation between poor nutritional status and diminished survival rates [50].

Crucially, malnutrition in HNC patients often coexists with sarcopenia and cachexia, conditions characterized by the loss of muscle mass and weight, respectively. This triad exacerbates patients’ vulnerability, adversely impacting their physical strength and overall treatment resilience. Nutritional interventions, therefore, aim not only to address caloric deficits but also to combat these muscle-wasting conditions, thereby preserving patients’ functional capacity and improving their response to cancer therapies.

**Figure 2:**
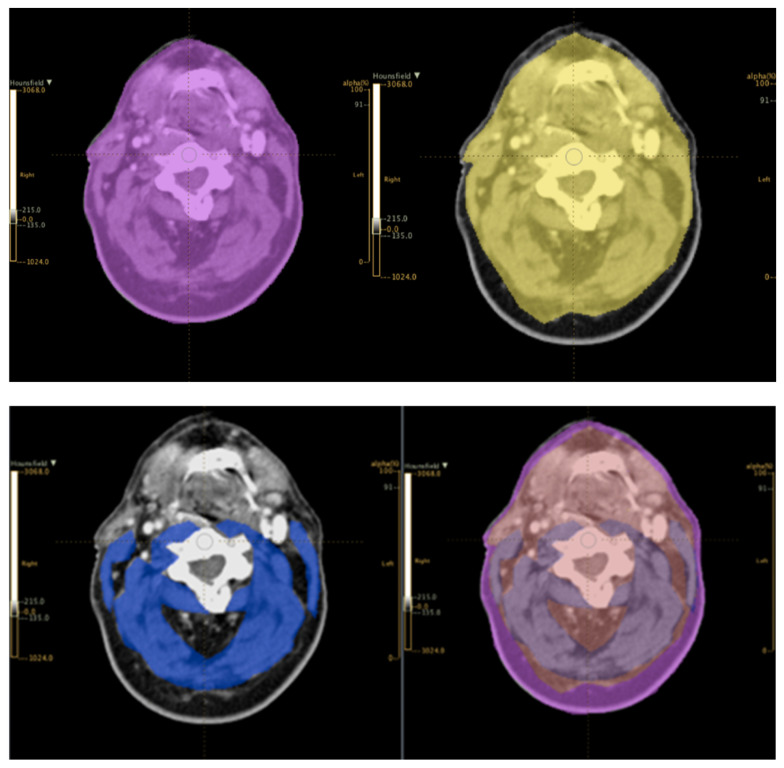
Example of a CT scan contouring at the level of C3. **Above**: contour of the skin profile on the **left**, contour of the subcutaneous fat on the **right**. **Bottom**: contour of the muscle profile on the **left**, three profiles shown simultaneously on the **right**.

Within the prehabilitation framework, nutrition serves not just as a sustenance measure but also as a strategic tool to bolster treatment tolerance and hasten recovery [51]. This approach necessitates a multifaceted strategy, beginning with rigorous nutritional assessments (e.g., the following laboratory tests before the start of treatment: blood count, renal and liver function, electrolytes, cholinesterase, lipid and iron tests, insulin, vitamins B12, D, and B9), followed by a close monitoring to promptly identify and address nutritional deficits during the therapies [10]. Tailored dietary interventions, which include diet texture modifications and the inclusion of nutrient-dense supplements, play a crucial role [52]. The efficacy of these interventions is significantly enhanced through the involvement of a multidisciplinary team comprising dietitians, oncologists, speech therapists, and other healthcare professionals [53]. There is no consensus on the ideal preoperative tool for “at-risk” surgical patients. Clinicians should select a tool that is suitable for their context, quick, easily interpreted, including components related to nutritional condition, stability, potential deterioration, and likely deficits due to disease progression [54]. Other complex entities such as sarcopenia, being underweight, or sarcopenic obesity might contribute to negatively influence the patients’ outcome and need to be assessed. Quantitative evaluations have been performed using anthropometric index and bioelectrical impedance analysis (BIA) but the increasing needs of nutritional support in terms of precise evaluation of the different compartments, i.e., muscle mass and fat mass, has led to the use of imaging, and especially radiological modality, to assess nutritional status. Nowadays, dual X-ray absorptiometry (DXA) is routinely used and computed tomography (CT scan), magnetic resonance imaging (MRI), and ultrasound (US) are expanding in use. DXA exploits the different attenuation of diverse tissues when exposed to X-rays to assess lean body mass (LBM), fat mass (FM), and bone mineral mass (BMM) with a whole-body examination. The radiation exposure is extremely low (0.1 mSv) [55]. The radiation exposure is higher for CT scans and nutritional indexes from CT scans are generally acquired as “fringe benefits” of the imaging follow-up in oncological patients. In the literature, the most widely diffused quantification system uses a slice passing through the vertebra L3 and the compartment assessment at that level as a predictor of several nutritional index and prognostic factors [55]. HNC patients in several settings do not undergo a CT scan evaluation of the abdomen and thus efforts have been put into correlating the same index and prognostic factors using the third cervical vertebra (C3) as the landmark [56]. In Figure 2, an example of a CT scan contouring at the level of C3 can be seen. A translation of MRI has been proposed; indeed, MRI is preferred over CT scans in HNC and the radiation exposure is lower [57]. The main challenges are related to the complexity of segmentation (mostly manual) and to the high variability of sequences and protocols, hampering the ability to standardize the results. The higher cost of MRI also reduces the possibility to add specific sequences for fat or water quantification (e.g., Dixon imaging), whose utility needs to be assessed. The ultrasound (US) ability to characterize tissue is related to the evaluation of the “speed of sound” that varies while crossing different tissues, and in addition to the absence of radiation, the low-cost and high availability are remarkable aspects [58]. Nevertheless, US application has been challenging and the results less consistent compared with other imaging modalities, mainly due to the lack of a panoramic view or a standardized measurement technique and protocols. US is still an active field of research and future progress may overcome several of these challenging aspects.

The nutritional status profile at diagnosis, in addition to the primary tumor site, stage of disease, and planned doses of CRT, are crucial to identify those patients who are candidates for intensive nutritional support by a naso-gastric feeding tube (NGT) or percutaneous endoscopic gastrostomy (PEG). According to international European Society of Parenteral and Enteral Nutrition (ESPEN) guidelines [59], routine enteral nutrition (EN) is generally not recommended during RT or CRT, even if a recent survey involving European clinicians demonstrated that prophylactic nutritional support is employed in 85% of HNC patients [60]. If needed, enteral nutrition has to be preferred over parenteral, either by NGT or by PEG [59]. PEG is preferred over NGT in patients with prognosis > 6 months, requiring > 1 month of nutritional support.

#### 3.2.3. Psychological Support

In addition to the feral anxieties that characterize all the neoplastic pathology, the major concern for HNC patients is the surgical and radiotherapeutic damage that may change their facial features and/or causes loss of function [61,62,63]. This induces heavy consequences on the QoL, especially on the psychological and relational sphere [64,65,66]. The aesthetic–functional damage related to demolitive interventions and the massive physical reactions to treatments induce experiences that may in fact belong to the category of trauma [67]. Total laryngectomy is an obvious example as it refers to a phase of development completely surpassed by the adult person, inducing a condition of regression that can call into the question their emotional development, profoundly touching the person’s identity [66]. Substance abuse or dependence are frequently reported, with frustration easily caused by stress, limited individual resources, and/or a tendency to passive regressive attitudes, factors that can make the patient’s adaptation to the challenges of the clinical path even more difficult [68,69]. In this context, a psychological evaluation in the initial phase can intercept the patient’s distress [70]. Constant attention needs to be maintained during the treatment to monitor the intervention itself, to redefine the objectives with the patient and to evaluate the psychological changes. This clinical evaluation must be able to detect some factors such as the degree of awareness, psychological and humoral reactivity to cancer, the expectations regarding therapies, the mental functioning of the patient (in order to identify any traits that may hinder the adaptation to the disease conditions), types and levels of distress, quality of social support, family relationships, and the presence of current or previous psychopathology [70]. The recognition or admission of tobacco and/or alcohol dependence is a priority to prevent any withdrawal syndromes [71]. These elements of clinical evaluation must be incorporated into a multidisciplinary consultation [69]. With the final aim of integrating the various phases of therapeutic interventions, an early multidisciplinary consultation is needed: communicative, psychological, humoral, and social care needs have to be considered [70]. Screening tools include the National Comprehensive Cancer Network Distress Thermometer, a simple Likert scale with patients asked to rate distress in the last week from 0–10, with a score ≥4/10 triggering referral for more in-depth assessment. Other short-form screening tools, such as the Patient Health Questionnaire-2, Distress Thermometer, and Hospital Anxiety and Distress Scale are reliable and acceptable to clinicians and patients. These tools have established cut-offs which indicate the need for more detailed assessment and, when indicated, psychological intervention [54]. However, the tendency of patients to implement mechanisms of denial and indifference to the effects and risks of the disease makes self-reporting questionnaires unreliable, as patients tend to perceive themselves defensively in an unrealistic way, minimizing difficulty and psychic suffering. In contrast, questionnaires may be applied when patients refuse psychological support. Since the literature indicates that these patients are unwilling to ask for psychological help, the main goal is to build a therapeutic alliance and to implement an intervention aimed not only at containing anxiety, but also to support patients in assuming their own responsibility during the decision-making process [72]. The objectives of this psychological work are to reduce preoperative and pre-treatment anxiety, to promote the patient’s awareness, and to inform, motivate, and reassure the patient about possible rehabilitation and/or prosthetic interventions regarding swallowing and phonation [73]. A higher awareness increases the patient’s perception of control as it induces a better acceptance of the clinical pathway. This grip of emotional and cognitive reality avoids the situation where the patient, overwhelmed by unexpected outcomes, cannot put in place the needed personal resources to deal with the remaining or ongoing problems. The last step of the patient’s psychological care path should include their participation in the construction of a network of connections with palliative care and, before that, with simultaneous care. The aim is to be able to guarantee the passage of those psychological and communicative needs in the institutional path to make patients’ experience in their last mile smoother.

Diagnosis and treatment of HNCs are emotionally and physically stressful periods for patients, with high risk of depression and suicide [74]. In recent years, a new psychological approach with cognitive behavioral intervention (CBI) has been implemented in the clinic, with the goal of improving functional impairment as well as post-treatment psychological distress [75]. CBI is a process by which patients learn to become experts of their own behavior, learning to examine their thoughts, recognize when negative thoughts are increasing, and then apply a number of strategies to alter those negative thoughts and emotions. In addition to CBI, other initiatives such as psychoeducation, meditation/mindfulness, group therapy, and telehealth initiatives have been implemented in the last 30 years [76]. Mindfulness, in particular, can be administered to HNC patients during active cancer treatments, as has been shown in a series with 19 patients [77]. A longer time spent meditating daily was associated with higher post-intervention mindfulness.

### 3.3. Prehabilitation and Impact on Cancer Progression

Multimodal prehabilitation could have a significant impact on the immune system, globally acting with the reduction of inflammatory processes and, as a consequence, influencing cancer progression. The immune system is very responsive to exercise. The immunomodulatory properties of physical activity can be summarized in four points: acute and chronic effects of exercise on the immune system; clinical benefits of the exercise–immune relationship; nutritional influences on the immune response to exercise; and the effect of exercise on immunosenescence. Preclinical studies with animal models demonstrated the growth-inhibitory effect of aerobic exercise on the tumor microenvironment, e.g., increased intratumoral immune cell infiltration (in particular, natural killer cells) resulting from epinephrine spikes and interleukin-6 release [78]. Preliminary results in different tumor types (e.g., breast and colorectal cancers) have proved that physical exercise stimulates the immune system response, even if with contradictory results [79,80]. Indeed, physical exercise could regulate anti- and pro-inflammatory cytokines, the activity of cytotoxic immune cells, and suppressor immune cells [81,82,83,84]. In breast cancer survivors, an aerobic and resistance training program has been associated with a significant serum levels reduction of c-reactive protein (CRP), interleukin (IL)-6, -8, and -10, and tumor necrosis factor (TNF)-α. Instead, physical exercises have significantly increased serum levels of IL-4 and heat shock protein (HSP) 70 [83]. Carbohydrate ingestion during exercise increases the level of glucose, insulin, and fructose, reduces the hormonal stress and, in turn, turns off the inflammation, through the reduction of circulating levels of neutrophils, monocytes, and cytokines [85]. Finally, physical exercise may influence the senescence program; exercise regulates the immune system, delaying the onset of immune senescence. In particular, exercise exerts its activity by reducing the numbers of exhausted/senescent T cells, increasing T cell capacity, neutrophil phagocytic activity, NK cell cytotoxic activity, and increasing leukocyte telomere lengths [86]. In addition, aerobic exercise behaviors (treadmill/wheel running) in tumor-bearing mice and rats appear to increase microvessel density, maturity, and perfusion causing reduced tumor hypoxia, increased blood-flow, and, ultimately, improved chemotherapy delivery [87]. The immunomodulatory effects induced by nutrition have been acknowledged for years [88]. In fact, the obese adipose tissue causes strong changes in production of cytokines and alteration of immune infiltrate [89]. For instance, the functions and proportions of macrophages are altered by obesity, which determines a switch from the anti-inflammatory phenotype (M2) to the pro-inflammatory phenotype (M1) of the macrophages in the adipose tissue and M1 macrophages produce oncogenic cytokines, such as TNF, IL-6, and IL-8 [89]. On the other side, fasting is associated with anti-inflammatory effects, for example the inhibition of IL-1β production by monocytes, which is restored only three hours after a meal [89]. Finally, it has been demonstrated that depression is associated with alterations in concentrations of various inflammatory markers, such as CRP, IL-6, IFN-γ, and TNF-α [90,91]. Furthermore, post-traumatic stress disorder (PTSD) is also characterized by inflammation dysregulation, determining changes in the levels of IL-6 and TNF-α [92]. Psychological support may have a potential role in immunomodulation. It is well known that chemotherapy also induces a general increase in inflammation causing an inflammatory environment [84]. Indeed, avoiding immune destruction and tumor-promoting inflammation are two of the well-known Hallmarks of Cancer proposed in 2000 [93]. Pro-inflammatory molecules, such as IL-6, TNF-α, and CRP induce both genetic and epigenetic alteration affecting all parts of the oncogenesis process, from initial single-cell mutations, to progression and systemic dissemination [88,94]. Finally, chronic inflammation in breast cancer patients is correlated with reduced overall survival and therefore could be a prognostic marker [95].

### 3.4. Active Clinical Trials in HNC Investigation Prehabilitation Interventions

Ongoing clinical trials in HNC, including prehabilitation program, are summarized in Table 2.

Up to now, the number of clinical trials investigating the role of prehabilitation in HNC patients is extremely limited. Almost all studies (three out of four) are focused on the prehabilitation program before surgical intervention, and only one includes patients who are candidates for CRT. In addition, a multimodal prehabilitation intervention has been included in one trial only (NCT05745558). These studies are characterized by different primary outcomes and this aspect jeopardizes the generation of evidence. Another weak point is the inclusion of clinical situations other than HNC; for example, trial NCT05745558 allows the participation of liver cancer patients while trial NCT04598087 has enrolled patients who are candidates for surgical resection for benign disease.

In Italy, we have recently opened a trial funded by the Ministry of Health through the PNRR resources (PNRR-TR1-2023-12377022; NCT06593639) involving five centers (ICS IRCCS Maugeri-Pavia, Fondazione IRCCS Policlinico San Matteo-Pavia, Azienda Ospedaliero Universitaria di Sassari, Istituto Nazionale Tumori IRCCS, Fondazione G. Pascale-Napoli, and CNAO, National Center of Oncological Hadrontherapy-Pavia). The trial investigates a multimodal prehabilitation program (combining physical exercise, nutritional intervention, and psychological support) carried out both before and during definitive cancer treatment for patients affected by locally advanced head and neck cancers. The primary objective of the study is to investigate the feasibility of the prehabilitation program.

### 3.5. Barriers to Implement Prehabilitation in Clinical Practice

The prehabilitation program can face barriers in clinical practice, limiting its diffusion. The feasibility of the intervention is the first issue: systematic reviews and meta-analysis in lung, breast, and colon cancer cohorts demonstrated clinically relevant recruitment, retention, and exercise adherence rates [96,97,98]. Adherence and completion of the intervention were similar in HNC patients, but they were lower in studies investigating nutritional and physical exercise [99]. The adherence to nutritional intervention was about 60% during the oncological treatment and about 75% after the treatment; for nutritional intervention, the completion rates varied between 70% and 92%; for exercise between 81% and 96%; while in the studies investigating combined nutritional and exercise interventions, they varied between 60% and 87%. The inclusion of the psycho-oncological intervention could be fundamental to improve the adherence to prehabilitation. More evidence is required to better explore how and where to engage patients in physical activity, e.g., performing exercises that are always supervised or consider also virtual monitoring and performing them inside or outside the hospitals. The lack of dedicated facilities inside the hospital represents a serious barrier in the implementation process of physical activity programs. In addition, not all prehabilitation interventions are reimbursed by the national health system, e.g., physical activity exercises. Finally, socioeconomic status (SES) represents a serious barrier to prehabilitation implementation. Since SES influences postoperative morbidity, in terms of worse surgical outcomes, e.g., longer LOS, higher rates of complications, and reduced overall survival, a retrospective analysis has evaluated the SES as a barrier in prehabilitation participation for patients who are candidates for surgery [100]. In this analysis, lower SES corresponded to lower participation rates and lower baseline functional capacity and thus worse candidates for surgery; at the same time, receiving prehabilitation improved the functional capacity (assessed by the 6 min walking test) and reduced LOS to a similar extent in patients with lowest and highest SES in comparison to their respective controls, highlighting the importance of implementing prehabilitation in order to reduce disparities among SES.

## 4. Conclusions and Future Directions

The integration of multimodal prehabilitation programs, including nutritional intervention, physical activity, and psycho-oncological support, is crucial in the care of patients with HNC. This holistic approach can significantly improve treatment tolerance, accelerate recovery, and enhance overall patient outcomes. Despite the promising benefits demonstrated by various studies, the implementation of prehabilitation in clinical practice remains limited due to several barriers, including lack of standardization, variability in patient engagement, and inadequate resources within healthcare systems.

To maximize the potential of prehabilitation, future research should focus on the development of standardized guidelines that can be universally applied across different healthcare settings. More randomized controlled trials are needed to establish the efficacy of specific interventions and identify the most beneficial combinations for HNC patients. Additionally, efforts must be made to overcome socioeconomic disparities that affect patient participation and to explore innovative ways to deliver prehabilitation, such as through telehealth platforms.

Collaboration among stakeholders, including healthcare providers, researchers, policymakers, and patient advocacy groups, is essential to integrate prehabilitation into routine cancer care. By fostering a multidisciplinary approach and promoting early intervention, we can better prepare patients for the challenges of cancer treatment, ultimately improving survival rates and quality of life for individuals with HNC.

## Figures and Tables

**Table 1:** Randomized clinical trials on prehabilitation in HNC patients treated with radiotherapy ± chemotherapy.

Study	Intervention Type	Description of Intervention	Population	Primary Outcome and Measure	Results
Kotz, 2013 [32]	Prophylactic swallowing exercises	Five intervention swallowing exercises (Effortful Swallow [27], two tongue base retraction exercises [28,29], the Super Supraglottic Swallow technique [30], the Mendelssohn maneuver [31]) initiated prior to RT and continued during CRT	26 patients receiving CRT; intervention group *n* = 13, control group *n* = 13	Differences in swallowing function assessed with Functional Oral Intake Scale (FOIS) [46] and Performance Status Scale for Head and Neck Cancer Patients (PSS-H&N) [47] at baseline, immediately after CRT, and at 3, 6, 9, and 12 months after CRT	No statistically significant differences in FOIS and PSS-H&N scores immediately after CRT; significantly better scores in intervention group at month 3 and 6 (median 3-month intervention score, 7 vs. median control score, 5 [*p* = 0.03]; median 6-month intervention score, 7 vs. median control score, 6 [*p* = 0.009]); no significant differences at months 9 and 12
Virani, 2015 [33]	Two different swallowing exercise regimens	Exercise group: Masako exercise (10 repetitions, seven sets daily), pharyngeal Squeeze exercise (10 repetitions, seven sets daily), shaker exercise (three sets daily). Swallow group: 34 swallows of saliva and/or water as needed in each of seven sets daily.	50 patients receiving RT ± CT; exercise group *n* = 26, repetitive swallow group *n* = 24	Differences in FOIS and percutaneous endoscopic gastrostomy (PEG) placements, compared to post-treatment and at 3 months afterwards	The exercise group eliminated significantly more PEG at 3 months compared to the swallow group (16% vs. 50%; *p* = 0.016). Among patients who received CRT, the exercise group had significantly less PEG tubes immediately post-treatment as well as 3 months post-treatment (35% and 10%), compared to the swallow group (69% and 50%) (*p* = 0.044 and 0.011)
Mortensen, 2015 [34]	Swallowing exercises	Range of motion drills to maintain and improve the range of motion of structures and muscle groups, and resistance exercises to strengthen the same muscles (tongue hold, gargle, tongue range of motion, jaw exercise, larynx range of motion, shaker exercise)	44 patients receiving RT ± CT; intervention group *n* = 22, control group *n* = 22	Differences in swallowing-related outcome: Swallowing Performance Status Scale (SPSS) score based on Modified Barium Swallow (MBS) examinations measured pre-treatment, 2, 5, and 11 months after the end of RT	No statistical difference of mean SPSS score was found between the two groups
Hajdú, 2022 [35]	Bimodal progressive resistance training (PRT) and swallowing exercises	Twice-weekly physiotherapy-led PRT, three-times weekly unsupervised swallowing exercise sessions, daily self-administered swallowing exercises. PRT program: six exercises covering lower limbs, upper body, and core in a fixed progression model based on maximum of repetitions. Swallowing exercises all or some of the following: reaching tongue back and forth, tongue to cheek, tongue to mouth corners, resistance to tongue, gargle, yawn, mouth opening, jaw side-to-side, jaw undershot, Valsalva, Shaker exercise, Mendelsohn maneuver, Masako maneuver, Effortful Swallow	240 patients receiving RT ± CT; intervention group *n* = 122, control group *n* = 118	Differences in penetration aspiration score (PAS), assessed with a fiberoptic endoscopic evaluation of swallowing (FEES), measured at end of treatment and 2, 6, and 12 months after	No statistical difference between groups for PAS
Loorents, 2014 [36]	Training with TheraBite^®^ Jaw Motion Rehabilitation System™	TheraBite utilizes passive motion and stretching of jaw musculature. Training program: five stretches, five times daily, 15 s each stretch, continued for 12 months after the end of RT	66 patients receiving RT ± CT; intervention group *n* = 33, control group *n* = 33	Differences in maximum interincisal openings (MIO), recorded at baseline, once a week during RT, 3, 6, and 12 months after RT	No significant differences in MIO at any time point between intervention and control groups
Lin, 2021 [39]	Combined aerobic, resistance and flexibility exercises	Moderate-intensity aerobic activity: treadmill (60–70% maximum heart rate); resistance training: Thera-Band or free weight (1–3 sets, 8–12 repetitions of exercises for large muscle groups, perceived exertion of moderate-hard on Borg scale); flexibility exercise: static stretching of large muscle groups. Intensity, volume, and frequency increased if participant was willing and able to progress. Started 3 days before first cycle of CT, continued during CT and for four weeks after first cycle	40 patients receiving CT; exercise group *n* = 20, control group *n* = 20	Body composition (measured with body composition monitor), muscle strength (30 s arm curl test, 30 s chair stand test), balance (timed up and go test), flexibility (back scratch test, hair sit-and-reach test), cardiovascular fitness (3 min step test) and HRQoL (EORTC QLQC30, EORTC-QLQ-H&N35); assessment at baseline and eight weeks following baseline	Significant difference between groups eight weeks after baseline in body composition (body fat percentage, *p* = 0.002; skeletal muscle percentage, *p* = 0.008), dynamic balance (*p* = 0.01), muscle strength (upper extremity, *p* = 0.037; lower extremity, *p* = 0.025) and HRQoL (*p* = 0.001)
Zhao, 2016 [40]	14-week functional resistance and walking program	Three sessions/week, 1 h/session; delivered at clinical research center during CRT and at home after CRT from week 8 to 14 with weekly telephone calls; functional resistance training, goal: three sets, 8–12 repetitions of each exercise; walking: multiple short duration continuous walking periods, e.g., 5 min six times during the day to achieve a total walking time of 30 min; home program, goal: minimum of 5 days/week, minimum of 30 min/day, at a moderate intensity (rate of perceived exertion, RPE = 11–13)	20 patients receiving CRT; MPACT group *n* = 11, control group *n* = 9	Muscle strength (measured with elbow flexion and knee extension strength and grip strength), functional mobility (gait speed over a 6 min distance and time to rise from a chair, walk 3 min away, and then return to the chair and sit down), self-reported QOL (Medical Outcomes Study (MOS) Short Form-36 (SF-36) and The six-item MOS Sleep Problem Index), and physician-reported concurrent CRT toxicity; assessments at baseline, 7 weeks, and 14 weeks	Trends statistically significant (*p*< 0.05) between groups in knee strength, mental health, head and neck QOL, and barriers to exercise
Samuel, 2019 [41]	11-week aerobic and active resistance program	7 weeks in the hospital during CRT and 4 weeks at home after CRT; five sessions/week; intensity 3–5/10 RPE on Borg’s scale; aerobic activity: brisk walking, 15–20 min; active resistance training for the major muscles of upper limb and lower limb done in two sets (one set = 8–15 repetitions)	148 patients receiving CRT; exercise group *n* = 58, control group *n* = 62	QoL measured with SF-36 and functional capacity measured with 6 min walking test	Significant improvement in the functional capacity (*p* < 0.001), quality of life (*p* < 0.001), and prevention of worsening of fatigue (*p* < 0.001) in the exercise group
Sandmael, 2017 [42]	Exercise (PRT) and nutrition intervention (ONS)	Exercise and nutrition intervention during RT (EN-DUR group) or exercise and nutrition intervention after RT (EN-AF group); EN-DUR group: intervention during RT, two PRT session/week, 30 min/session, 3–4 sets, 6–12 repetitions at an outpatient training facility and ONS (minimum of one nutritional drink Monday-Friday, 2 deciliters and 350 kilocalories; EN-AF group: 3-week intervention 2 to 4 weeks after the end of RT, three PRT sessions/week, 45 min/session, 3–4 sets, 6–12 repetitions at a rehabilitation center, ONS (the same as EN-DUR group), and nutritional counseling	41 patients receiving (C)RT; EN-DUR group *n* = 20, EN-AF group *n* = 21	Feasibility by tracking recruitment, attendance, adherence, and attrition rates	82% patients agreed to participate. EN-DUR group: attendance, 90%, adherence to PRT, 91%, to ONS, 57%. EN-AF group: attendance, 52% adherence to PRT, 94%, to ONS, 76%
Capozzi, 2016 [43]	12-week lifestyle intervention and progressive resistance training	12-week immediate lifestyle intervention (ILI) group or delayed lifestyle intervention (DLI) group, started after 12 weeks; five components of lifestyle intervention: physician referral and clinic support, health education, behavior change support, individualized exercise program (four sessions/week, PRT program, two sets of eight exercises), social support	60 patients receiving (C)RT; ILI group *n* = 31, DLI group *n* = 29	Body composition (lean body mass, body mass index, body fat)	No significant difference
Cereda, 2018 [44]	Nutritional counseling with or without ONS	Nutritional counseling: personalized dietary prescription tailored on personal eating patterns and preferences and weekly dietitian consultation; ONS group: two bottles/day (250 mL) of a ready-to-use energy-dense, high-protein, omega-3 enriched oral formula providing 500 kilocalories, 23 g of protein and 1.9 g of omega-3 fatty acids	159 patients receiving RT; intervention group *n* = 78, control group *n* = 81	Change in body weight measured with a calibrated scale with a stadiometer and BMI; assessments at baseline, end of RT, 1 month and 3 months after the end of RT	Nutritional counseling with ONS is associated with smaller loss of body weight than counseling alone (mean difference, 1.6 kg; *p* = 0.006)
Roussel, 2017 [45]	Intensive nutritional care (INC)	Six individualized meetings with the dietitian at home. Two consultations during RT, last four at the end of RT and then 2 months later; all patients received nutritional care (at least two outpatient consultations with a dietitian; nutritional adjustments with regular foods, oral supplements, or tube feeding if indicated)	87 patients receiving (C)RT; INC group *n* = 43, control group *n* = 44	Change in QoL, using EORTC-QLQ-C30, assessed at baseline and 3 months after the end of the treatment	No significant difference in EORTC-QLQ-C30 between the two groups at baseline and 3 months after RT

**Table 2:** Active clinical trials on prehabilitation in HNC.

ClinicalTrials.gov ID	Randomization	Treatment	Prehabilitation Intervention	Primary Outcome	Measure Description of Primary Outcome
NCT05418842	Yes	CRT	Exercise training	Functional capacity	Six-minute walking test
NCT05745558	No	Surgery	Multimodal (training and nutritional, smoking cessation, and psychosocial counselling)	Feasibility	Self-designed questionnaire score; number of sessions that pts participate; pts willing to participate
NCT04598087	No	Surgery	Aerobic and resistance training	Quality of life	Functional Assessment of Cancer Therapy-Head and Neck (FACT-H&N)
NCT06079697	Yes	Surgery	Exercise training	Postoperative mobility	Daily step counts on the Fitbit^®^ or personal wearable device
